# Environmental Justice Research: Contemporary Issues and Emerging Topics

**DOI:** 10.3390/ijerph13111072

**Published:** 2016-11-01

**Authors:** Jayajit Chakraborty, Timothy W. Collins, Sara E. Grineski

**Affiliations:** Department of Sociology & Anthropology, University of Texas at El Paso, El Paso, TX 79968, USA; twcollins@utep.edu (T.W.C); segrineski@utep.edu (S.E.G)

**Keywords:** environmental justice, social inequality, health, air pollution, water pollution, flood, food, energy, green space, sustainability

## Abstract

Environmental justice (EJ) research seeks to document and redress the disproportionate environmental burdens and benefits associated with social inequalities. Although its initial focus was on disparities in exposure to anthropogenic pollution, the scope of EJ research has expanded. In the context of intensifying social inequalities and environmental problems, there is a need to further strengthen the EJ research framework and diversify its application. This Special Issue of the *International Journal of Environmental Research and Public Health (IJERPH)* incorporates 19 articles that broaden EJ research by considering emerging topics such as energy, food, drinking water, flooding, sustainability, and gender dynamics, including issues in Canada, the UK, and Eastern Europe. Additionally, the articles contribute to three research themes: (1) documenting connections between unjust environmental exposures and health impacts by examining unsafe infrastructure, substance use, and children’s obesity and academic performance; (2) promoting and achieving EJ by implementing interventions to improve environmental knowledge and health, identifying avenues for sustainable community change, and incorporating EJ metrics in government programs; and (3) clarifying stakeholder perceptions of EJ issues to extend research beyond the documentation of unjust conditions and processes. Collectively, the articles highlight potentially compounding injustices and an array of approaches being employed to achieve EJ.

Environmental justice (EJ) research seeks to document and redress the disproportionate environmental burdens and benefits associated with social inequalities. Although its initial focus was on anthropogenic pollution, the scope of EJ research has expanded significantly in recent years to encompass other phenomena—for example, access to healthful food and climate change—with disparate negative impacts on particular social groups. Dimensions of social inequality examined have expanded beyond race and socioeconomic status to focus to some degree on ethnicity, immigration status, gender, sexual orientation, age, as well as intersections between dimensions of inequality. In the context of intensifying social inequalities and environmental problems, there is a need to further strengthen the EJ research framework and diversify its application. This Special Issue of the *International Journal of Environmental Research and Public Health (IJERPH)* incorporates 19 articles that collectively advance EJ scholarship in conceptual, methodological, and empirical terms.

These articles demonstrate how the scope and purpose of EJ research have broadened significantly in recent years and continue to expand in new directions, both topically and geographically. Several articles in this Special Issue break new ground by extending the EJ research framework to consider emerging issues such as energy [1,2], food [3], drinking water [4,5], flooding [6,7], sustainability initiatives [8,9], and gender dynamics [10], including EJ concerns in Canada [5,11], the UK [12], and Eastern Europe [13]. Finley-Brook and Holloman [1] explore the EJ implications of energy production in the U.S. Their study demonstrates how the transition from high carbon energy sources such as coal and oil contribute to environmental injustices, and proposes priorities for a new energy justice research agenda that combines advocacy, activism, and academics. Kyne and Bolin [2] focus on nuclear hazards associated with both the U.S. weapons programs and civilian nuclear power. Their article argues that nuclear power plants, uranium mining, and waste disposal raise a variety of EJ issues that encompass distributive, procedural, recognition, and intergenerational justice. Carrel et al. [3] examine the EJ impacts of animal feeding operations in Iowa, USA. Their findings underscore the need to understand the structural, political, and economic factors that create an environmentally unjust landscape for swine production in the U.S. Midwest. Galway [4] investigates access to safe and reliable drinking water in First Nations communities in Ontario, Canada, based on drinking water advisory data. The study highlights the prevalence of drinking water advisories as a growing problem that needs to be addressed. Campbell et al. [5] focus on the governmental failures in treating the municipal water system that led to the poisoning of hundreds of children and adults in Flint, Michigan, USA, and discuss how such tragic events can be prevented in the future. Maldonado et al. [6] examine if Hispanic immigrants are disproportionately exposed to flood hazards compared to other racial/ethnic groups in the Houston and Miami metropolitan areas, USA, based on household-level survey data. Their divergent findings for these two urban areas suggest that future EJ research on flooding should distinguish between Hispanic subgroups based on nativity status and other local contextual factors. Muñoz and Tate [7] focus on the EJ consequences of disaster recovery, based on a case study of three communities in Iowa, USA, that were affected by severe flooding in 2008. Their analysis of the two federal programs that funded property acquisitions indicated that households in socially vulnerable areas were less likely to obtain full financial compensation and endured longer waiting periods before receiving acquisition funds. Jennings et al. [8] examine another emerging issue in EJ research: advancing sustainability by ensuring that urban ecosystem services and related health benefits are equally distributed across all population groups. Their article integrates complementary concepts from multiple disciplines to illustrate how cultural ecosystem services from urban green spaces are associated with equity and social determinants of health. Hornik et al. [9] explore how people conceptualize the connection between EJ and sustainability, based on analyzing stakeholder perspectives in Milwaukee, WI, USA. Bell [10] addresses an important gap in prior EJ research by providing a gender perspective and exploring women’s experience of EJ, based on a review of the existing literature and her own prior experiences as a scholar and activist. Bell’s analysis confirms that women tend to experience inequitable environmental burdens and are less likely than men to have control over environmental decisions, both of which lead to disproportionate health impacts.

In addition to broadening the scope of EJ scholarship by exploring these new frontiers, our Special Issue contributes to three specific research themes: (a) documenting connections between unjust environmental exposures and health impacts; (b) promoting and achieving EJ; and (c) clarifying stakeholder perceptions of EJ issues. These themes and related articles are described below.

*Documenting connections between unjust environmental exposures and health impacts*: As the EJ framework has expanded in new directions, recent research has emphasized the need to examine health outcomes and health disparities associated with exposure to environmental hazards, thus extending EJ to environmental health justice. Several articles in this Special Issue advance environmental health justice scholarship by documenting linkages between unequal environmental exposure and adverse health impacts associated with unsafe infrastructure and homes [5,14], substance use and addiction [15], and children’s obesity and academic performance [16]. Campbell et al. [5] provide a detailed assessment of the recent drinking water crisis and lead poisoning in Flint, USA. In addition to describing how this tragedy happened and why socially disadvantaged populations are at particularly high risk for lead exposure, Campbell et al. discuss how childhood lead exposure and Flint-like events can be prevented from occurring in the future. Mankikar et al. [14] examine whether participation in a two-month long environmental education intervention program reduces exposure to homebased environmental health hazards and asthma-related medical visits. Their home intervention program in southeastern Pennsylvania, USA, focused on low-income households where children had asthma, were at risk for lead poisoning, or faced multiple unsafe housing conditions. Cleaning supplies (e.g., a microfiber cloth, soap), safety supplies (e.g., CO detector, fire alarm) and pest management tools (e.g., caulk, roach bait) were provided along with educational materials and face-to-face instruction. Their findings indicate that low-cost comprehensive home interventions are effective in reducing environmental home hazards and improve the health of asthmatic children in the short term. Mennis et al.’s [15] review article seeks to extend EJ research by including environmental factors influencing substance use disorders—one of the most pressing global public health problems. They demonstrate why inequities in risky substance use environments should be considered as an EJ issue and conclude that future research needs to examine where, why, and how inequities in risky substance use environments occur, the implications of such inequities for disparities in substance use disorders and treatment outcomes, and the implications for tobacco, alcohol, and drug policies as well as prevention and treatment programs. Clark-Reyna et al. [16] focus on chemicals known as metabolic disruptors that are of specific concern to children’s health and development. Their article examines the effect of residential concentrations of metabolic disrupting chemicals on children’s school performance in El Paso, Texas, USA. Results indicate that concentrations of metabolic disruptors are significantly associated with lower grade point averages directly and indirectly through body mass index. Findings from this study have important implications for future EJ research and chemical policy reform in the U.S.

*Promoting and achieving EJ*: While EJ scholars often focus on describing the injustices experienced by socially disadvantaged communities, several articles in this Special Issue direct attention toward efforts to achieve EJ through implementation of interventions to improve environmental knowledge and health [14,17], identification of avenues for sustainable and just community and societal change [1,8,9,13], and incorporation of EJ metrics in government programs [12]. In the area of interventions, Ramirez-Andreotta et al. [17] examine parental perceptions of the “report back” process after an exposure assessment. Results showed that parents coped with their challenging circumstances using data and that they made changes to reduce children’s exposure to contaminants. The findings suggest that providing information to EJ community members could be an effective strategy to reduce exposure, when immediate wider scale remediation is not possible. While Mankikar et al. [14] was summarized above, what is relevant here is that low income communities disproportionately face challenges from poor quality housing, especially renters. The promise of the type of intervention conducted by Mankikar et al. for achieving EJ is that it works to improve the environmental health of children. In terms of identifying avenues for change, Hornik et al. [9] examine stakeholder beliefs about how positive change should be made to ameliorate injustices related to water pollution in Milwaukee, WI, USA. In order to work towards EJ, the authors argue that is important to build mutual understanding among stakeholders and acknowledge the potential for complex interactions across scales of governance in order to mitigate conflicts. Related to avenues for achieving EJ, Finley-Brook and Holloman [1] emphasize the importance of involving communities in the participatory design of solutions and fairly distributing benefits. The energy case studies they review suggest that empowering approaches are feasible, but also highlight the potential for conflict between what is “green” and what is “just”. Petrescu-Mag et al. [13] explore EJ issues in a Roma community in Romania beset by environmental challenges associated with a landfill. Researchers engaged community residents in discussions about potential action options, and residents strongly preferred improving local on-site living opportunities at the dump. An examination of the process of selecting this option suggests that negotiations among stakeholders are required in order to begin to address environmental injustices. Jennings et al. [8] argue that it is critical for all communities to have access to cultural ecosystem services that influence social determinants of health in order to achieve health equity and promote physical and psychological well-being. Taking a different approach, Fairburn et al. [12] trace the development and diffusion of indices of multiple deprivation (IMD). EJ scholars have impacted public policy through the incorporation of environmental data into IMD in England, Wales, and Northern Ireland, and evidence suggests that IMD are potential catalysts for EJ as they enable decision-makers to make more equitable decisions.

*Clarifying stakeholder perceptions of EJ issues*: The EJ research framework has focused on objectively documenting conditions and processes that constitute environmental injustices. Based on this materialist foundation, less emphasis in EJ research has been placed on people’s subjectivities. Several articles in this Special Issue advance EJ research by examining and clarifying stakeholder subjectivities regarding EJ issues [9,11,18,19], which extends the research framework beyond the documentation of unjust conditions and processes. In Hornik et al.’s [9] study, which clarifies community group perceptions of EJ in the context of water sustainability initiatives in Milwaukee, WI, USA, stakeholders shared similar perspectives on environmental injustice as an everyday experience. However, they had divergent perspectives on how environmental injustices are produced and most effectively redressed, which has implications for promoting initiatives for EJ and sustainability. Teixeira and Zuberi [18] examine neighborhood perceptions of environmental health hazards among black youth in Pittsburgh, PA, USA. Youth identified the intersection of race and poverty, poor waste management, housing abandonment, and crime as salient neighborhood environmental concerns, and understood correctly (based on the authors’ analysis of secondary spatial data) that black vs. white neighborhoods in the city are characterized by unequal environments. Findings suggest that environmental conditions provide clearly recognizable indicators of injustice for youth, and, furthermore, that youth interpret the lack of response to unjust conditions to imply that no one cares. Songsore and Buzzelli [11] examine the role of Ontario, Canada media in amplifying people’s perceptions of wind energy development (WED) health risks and injustices. Scientific evidence for negative health effects of wind turbines is contested, yet provincial media legitimated concerns about serious health impacts, which amplified public health risk perceptions and aroused claims of procedural injustice regarding the lack of community participation in Ontario’s WED process. Findings highlight the importance of media in shaping perceptions of environmental injustice, and reveal how public perceptions of injustice may be cultivated to impede societal transitions toward renewable energy sources. Ard et al. [19] use multilevel models in a US national study of the roles of neighborhood social capital and exposure to industrial air pollution in explaining the racial gap in self-rated health between black, Hispanic, and white individuals. They found that individuals’ feelings of trust in neighbors of different social standing and perceptions of political empowerment largely accounted for lower self-rated health among African Americans (and partially accounted for it among Hispanics) relative to whites, while exposure to industrial air pollution was statistically irrelevant. Results suggest that people’s perceptions of well-being may be shaped largely by their social contexts, and that harmful environmental exposures may not always be of paramount importance in shaping those perceptions. Taken together, these articles underscore how people’s subjectivities deeply matter: they influence which phenomena are contested as EJ issues and condition possibilities for redressing environmentally unjust arrangements.

The wide array of environmental health hazards, communities, and countries represented in this Special Issue reflect the expanding scope and purpose of EJ research, which has broadened and transformed significantly in recent years. The articles cover topics ranging from energy, food, water, obesogenic chemicals, landfills, and greenspace. They document connections between unjust environmental exposures and health impacts; provide ideas for how to promote and achieve EJ; and clarify stakeholder perceptions of EJ issues. In doing so, the Special Issue illustrates the existence of multiple and compounding marginalities, but also the wide variety of approaches being employed to achieve EJ, in its many diverse forms.
